# Remarkable Case of Alveolar Abscess and Ulceration of the Fang

**Published:** 1839-09

**Authors:** Augustus Woodruff Brown


### REMARKABLE CASE OF ALVEOLAR ABSCESS AND ULCERATION OF THE FANG.

[ BY AUGUSTUS WOODRUFF BROWN. ]

In the summer of 1838, a lady of Newtown, Long Island, called on me professionally, and showed me an open ulcer directly under the centre of the chin, and at least an inch from its anterior apex. This ulcer had been in a state of discharge for fifteen years excepting that it had been closed three times during that period for about three months at a time.

This ulcer had been pronounced by several physicians, to have no connexion with any of the teeth. In fact all the lady's front lower teeth excepting one, were in a perfectly healthy condition. This one was a central incisor, exactly over the diseased part. Remedies had been applied by many medical practitioners both in town and country to no valuable purpose, until I extracted the diseased incisor, after which, in twelve days the abscess was completely healed, and remains so to the present time.

The tooth when removed was found to be black and necrose in all its bony structure ; nothing but the enamel retaining its natural appearance.

The most remarkable feature in this case, was the fact that the *pus* from the ulcerated fang, had penetrated through the alveolus to the inner surface of the maxillary bone exactly behind the symphysis; thence it found its way downwards through the integuments, till it pierced the skin below as already described.

---

We are happy in being able to inform the profession that I. I. Greenwood, Esq. of this city, has placed in the hands of the Publishing Committee, the Autobiography of his father, with a copperplate likeness, of which we shall avail ourselves in the next number of this Journal.

